# Response of *Microtermes mycophagus* (Isoptera: Termitidae) to twenty one wood species

**DOI:** 10.7717/peerj.1132

**Published:** 2015-08-18

**Authors:** Naeem Iqbal, Hafiz Azhar Ali Khan, Shafqat Saeed

**Affiliations:** 1 Department of Entomology, Bahauddin Zakariya University, Multan, Pakistan; 2 Department of Plant Protection, Faculty of Agricultural Sciences, Ghazi University, Dera Ghazi Khan, Pakistan; 3 Institute of Agricultural Sciences, University of the Punjab, Lahore, Pakistan

**Keywords:** Feeding response, *Microtermes mycophagus*, Monitoring and baiting, Sapwood

## Abstract

The responses of termite species to bait depend upon the quality of the food used in the stations. Woods are the most common food sources for termites but different termite species behave differently to different wood species and types. The knowledge of the preference status of different wood species to a termite species helps in effective monitoring and baiting program. The current study was carried out to evaluate the preference of 21 wood species to the termite, *Microtermes mycophagus* in the field by no-choice and choice feeding tests. The results indicated silk cotton tree and sacred fig woods as the most preferred wood species with mean mass losses of 71.21 ± 5.09% and 68.38 ± 7.27% in no-choice test and 95.02 ± 1.65% and 91.69 ± 2.07% in choice tests, respectively. White cedar was the least preferred wood species with mean mass losses of 7.49 ± 1.64% and 13.92 ± 1.89% in no choice and choice feeding tests, respectively. Based on present studies, sapwood of silk cotton tree and sacred fig may be used in effective monitoring and baiting program against *M. mycophagus*.

## Introduction

Termites are the key pests of wood and wood products in tropical and sub tropical areas of the world (Lee, Ngee & Lee, 2003). The most recent strategy to suppress or eliminate termite infestations in the urban environment is “baiting” (Evans, 2010; Evans & Iqbal, 2015). This technique employs the use of non repellent and slow acting poison that is eaten and distributed by the foraging workers throughout the colony (Esenther & Beal, 1979; Grace et al., 1996). But baiting took several months to eliminate termite infestations compared to all other termite control methods (see Evans, 2010). However, termite control through baiting can be accelerated if baits are discovered quickly and more numbers of workers forage towards the bait station. These foraging workers will take more bait back to the colony, hence quick death of the colony members. Maximum numbers of workers can be trapped towards stations by placing adequate amount of highly palatable material in appropriate stations (Evans & Gleeson, 2006).

The first thing that can affect bait discovery and the termite foragers is the palatability of the food material used (Swoboda, 2004). Woods are the most common food sources for termites to eat in the environment so these could be economical and feasible materials to be used for termite traps. However, woods differ in their properties and can affect termite preference. Some wood species that are highly preferred by a termite species over others (Waller, Jones & La Fage, 1990; Morales-Ramos & Rojas, 2001), might be due to factors like wood hardness, moisture in the wood, wood decayed by fungus, allelochemicals in the woods, temperature, number of termite species in the area and their density, caste composition, and soldier proportion (Becker, 1969; Watson, Ruyooka & Howick, 1978; Sornnuwat et al., 1995; Fei & Henderson, 2002; Ngee et al., 2004; Arango et al., 2006; Gautam & Henderson, 2011; Little et al., 2013).

The genus *Microtermes* belongs to family Termitidae and subfamily Macrotermtinae. The species belonging to this genus are fungus growers and have a mutualistic relationship with the fungus. *Microtermes mycophagus* (Desneux) is a fungus growing termite and has been reported as a desert termite is Pakistan by Akhtar & Sarwar (1993). This species has been recorded from various districts of Punjab such as Bhawalpur, Multan, Muzaffargarh, Mianwali, Khanpur, Lahore and others (Sheikh, Bano & Akhtar, 2005; Manzoor & Mir, 2010; Iqbal & Saeed, 2013). It causes damage to the agroecosystem and residential wood structures (Iqbal & Saeed, 2013).

Keeping in view the importance of the quality of the food in monitoring and baiting studies, the current study was conducted to find out the most preferred native wood species for future baiting strategy to manage the infestation of *M. mycophagus* termite in Pakistan. In the present study, we used sapwood because it is soft compared to heartwood and can be efficiently used in baiting (Bultman, Beal & Ampong, 1979; Kasseney, Deng & Mo, 2011). Although sapwoods are soft and easy to chew, they differ in their preference to termites (Morales-Ramos & Rojas, 2001; Arango et al., 2006; Indrayani et al., 2007). So, in the feeding preference trial, 21 sapwood species were evaluated for their preference to *M. mycophagus*.

## Materials and Methods

### Test site

The tests were conducted in five year old tree plantations located near the Department of Entomology Block, Bahauddin Zakariya University, Multan, Pakistan where many trees are affected by *M. mycophagus*. The tests were carried out at sites of known termite (*M. mycophagus*) activity determined by burying survey stakes in the area.

### Feeding preference of *M. mycophagus* to wood species

A total of 21 wood species were evaluated for their preference to *M. mycophagus* (Table 1). Small pieces (10 by 1.5 by 1.5 cm) of wood were prepared and oven dried at 60 °C for two days. After that they were removed from the oven, cooled to room temperature and weighed on a balance. The preference of the wood species was tested in the field with no-choice and choice tests by following the methodology of Ngee et al. (2004).

#### No-choice feeding test

For no-choice tests, six sets of each wood species with four pieces in each set were prepared by tying with nylon cord. There were a total of 126 sets for all the wood species combined. Each set was placed in a plastic container (15 cm high by 5 cm in diameter) and buried in a grid pattern 1 m from each other. These sets were left in the field for 45 days after which they were removed and brought to the Entomology laboratory for re-weighing to calculate the mass of wood removed from each set. Before weighing the wood pieces were washed with water and oven dried as described above.

#### Choice feeding test

For choice tests, 10 sets of each wood species were evaluated by following the methodology as for the no-choice test. A total of 21 sets (one set of each wood species) were placed in a plastic container (30 cm in diameter by 15 cm high). Care was taken to tag each wood set permanently so that they were easy to distinguish at the end of the trial. This trial was terminated after 45 days. The samples were washed and reweighed as in the no-choice test.

After washing and weighing the wood pieces in both no-choice and choice trials, they were also visually rated according to the method described by American Standards for Testing and Materials (ASTM, 1984). This include 0 = no attack, 1 = slight superficial attack, 2 = superficial to medium attack (not deep inside), 3 = heavy attack (to penetration), 4 = very heavy attack (almost collapsed) to completely consumed. Response to wood species in term of preference was assessed by the amount of mass loss and visual rating results with more consumption from the preferred woods.

### Data analyses

The amount of wood consumed was determined by the difference in weight observed in the blocks before and after exposure to *M. mycophagus*. Data in mass loss (%) were subjected to analysis of variance (ANOVA), and their means were separated by Tukey’s HSD by using Statistix version 8.1 (Analytical software, 2005).

## Results

### Feeding preference of *M. mycophagus* to wood species

#### No-choice feeding test

**Figure 1:**
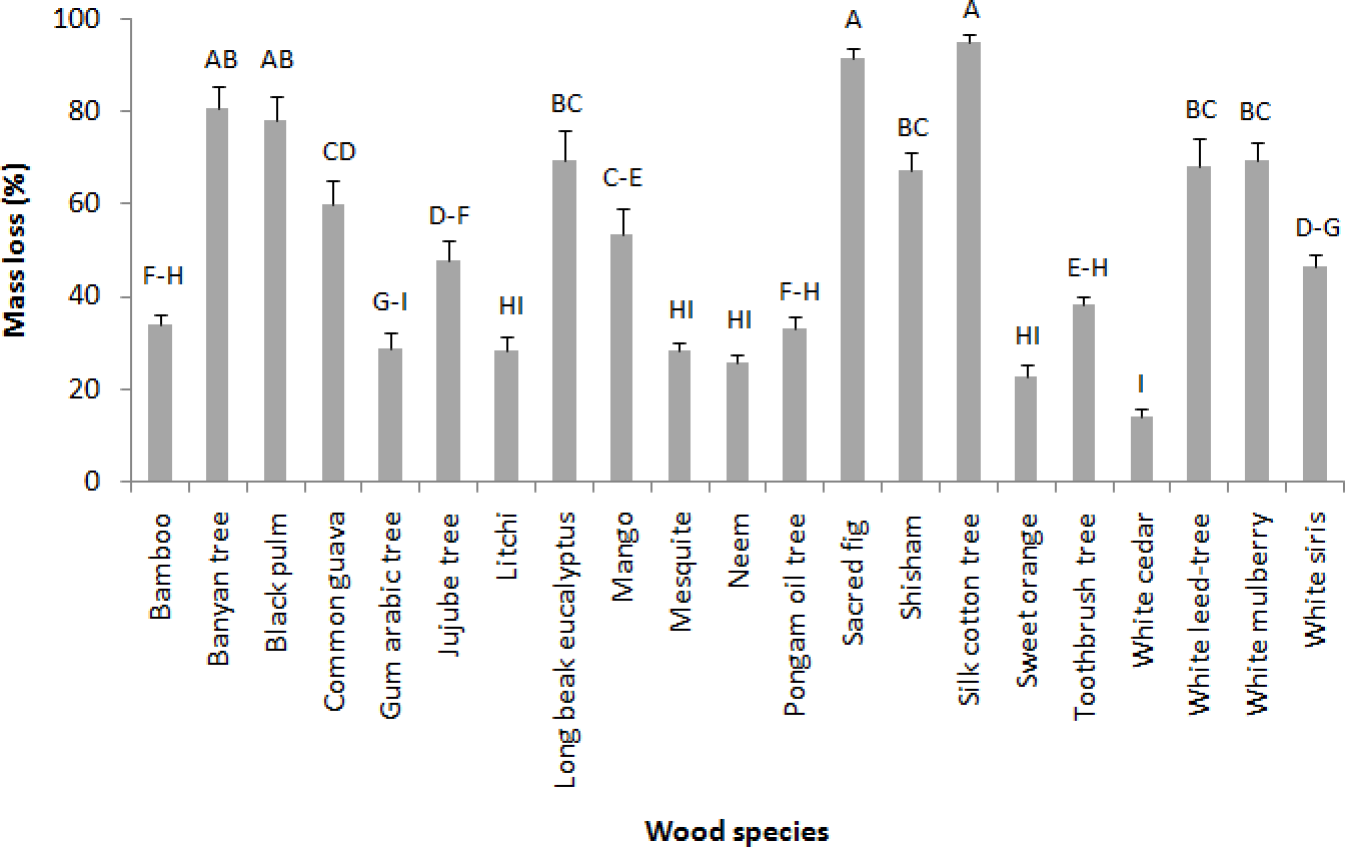
Mean percent mass loss of various wood species after 45 days no-choice feeding test against *M. mycophagus*. Means sharing the same letters are not significantly different (*P* > 0.05; Tukey’s HSD, Statistix 8.1).

The results of average mass losses from 21 wood species in the no-choice trial are shown in Fig. 1. The statistical analysis revealed significant differences in the mass losses of wood species (*F* = 24.57; df = 20, 100; *p* = 0.00). Silk cotton tree and sacred fig wood species were significantly preferred with the mass losses of 71.21 ± 5.09% and 68.38 ± 7.27%, respectively. However, white cedar, sweet orange, pongam oil tree, and litchi were regarded as significantly less preferred wood species with 7.49 ± 1.64%, 8.48 ± 1.84%, 10.11 ± 1.09% and 10.66 ± 2.65% mass losses, respectively (Fig. 1). Visual rating analysis results of all the wood species in the no-choice experiment are given in Table 1. The wood species that suffered heavy attack (deep to penetration) were silk cotton, sacred fig, and banyan trees, respectively. On the other hand, common guava, jujube tree, mango, shisham, whitesiris, black pulm, white leed-tree and long beak eucalyptus were respectively visually rated to the category of “superficial to medium attack (not deep inside)”. The species which had “slightly superficial attack” include white cedar, sweet orange, pongam oil tree, gum arabic tree, litchi, bamboo, neem, toothbrush tree, mesquite and white mulberry (Table 2).

**Table 1:** Wood species tested for preference by *M. mycophagus.*

English name	Local name	Botanical name
Bamboo	Baans	*Bambusa vulgaris* Schrad. ex J.C. Wendl.
Banyan tree	Bargad	*Ficus bengalensis* L.
Black pulm	Jaman	*Eugenia jambolana* Lam.
Common guava	Amrood	*Psidium guajava* L.
Gum arabic tree	Kikar	*Acacia nilotica* (L.) Willd. Ex Delile
Jujube tree	Bair	*Ziziphus jujube* Miller.
Litchi	Lichi	*Lichi chinensis* Sonn.
Long beak eucalyptus	Sufaida	*Eucalyptus camaldulensis* Dehnh.
Mango	Aam	*Mangifera indica* L.
Mesquite	Jungli kikar	*Prosopis alba* G.
Neem	Neem	*Azadirachta indica* A. Juss.
Pongam oil tree	Sukhchain	*Pongamia pinnata* L. Pierre
Sacred fig	Peeple	*Ficus religiosa* L.
Shisham	Shisham	*Dalbergia sissoo* Roxb. ex DC.
Silk cotton tree	Simbal	*Bombax malabaricum* L.
Sweet orange	Kino	*Citrus sinensis* L. Osbeck.
Toothbrush tree	Jaal	*Salvadora persica* Wall.
White cedar	Bakain	*Melia azedarach* L.
White leed-tree	Ipil Ipil	*Leucaena leucocephala* (Lam.) de Wit.
White mulberry	Shehtoot	*Morus alba* L.
White siris	Shreen	*Albezia procera* Roxb. Benth.

**Table 2:** Mean visual rating of various wood species against *M. mycophagus*.

**Wood species**	Visual rating ± SEM	
Key	No-choice test	Choice test
Bamboo	1.2 ± 0.2	2.0 ± 0.1
Banyan tree	2.8 ± 0.2	3.4 ± 0.2
Black pulm	2.2 ± 0.2	3.4 ± 0.2
Common guava	1.8 ± 0.2	2.9 ± 0.2
Gum arabic tree	1.0 ± 0.0	1.9 ± 0.2
Jujube tree	1.8 ± 0.2	2.8 ± 0.1
Litchi	1.0 ± 0.0	1.7 ± 0.2
Long beak eucalyptus	2.3 ± 0.3	3.1 ± 0.2
Mango	2.0 ± 0.0	2.5 ± 0.2
Mesquite	1.3 ± 0.2	2.1 ± 0.1
Neem	1.2 ± 0.2	1.8 ± 0.1
Pongam oil tree	1.0 ± 0.0	2.1 ± 0.1
Sacred fig	2.8 ± 0.1	3.8 ± 0.1
Shisham	2.0 ± 0.0	3.0 ± 0.0
Silk cotton tree	3.2 ± 0.1	3.9 ± 0.1
Sweet orange	1.0 ± 0.0	1.6 ± 0.2
Toothbrush tree	1.3 ± 0.2	2.1 ± 0.1
White cedar	1.0 ± 0.0	0.8 ± 0.1
White leed-tree	2.3 ± 0.2	3.0 ± 0.1
White mulberry	1.5 ± 0.2	3.1 ± 0.1
White siris	2.0 ± 0.0	2.8 ± 0.1

#### Choice feeding test

**Figure 2:**
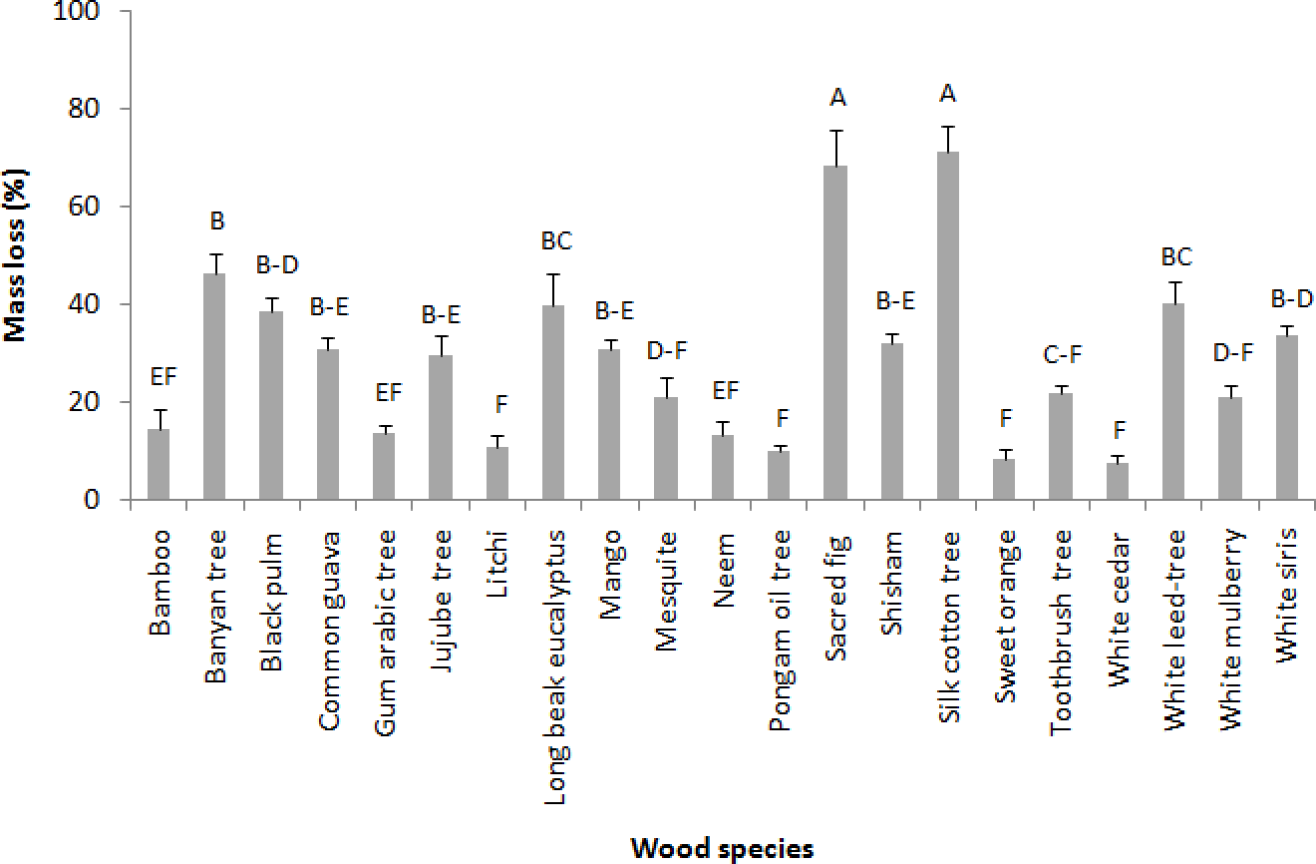
Mean mass loss (%) of various wood species after 45 days choice feeding test against *M. mycophagus*. Means sharing the same letters are not significantly different (*P* > 0.05; Tukey’s HSD, Statistix 8.1).

Choice feeding tests indicated almost similar results as in no-choice feeding test. The wood species differ significantly in term of preference to *M. mycophagus* in choice tests (*F* = 47.51; df = 20, 180; *p* = 0.00). Significantly highest preference was observed for the silk cotton tree and sacred fig with mass losses of 95.02 ± 1.65% and 91.69 ± 2.07%, respectively. White cedar differed significantly from all other wood species and was regarded as the least preferred wood species with very less mass loss of 13.92 ± 1.89% in 45 days (Fig. 2). Based on the visual rating analysis, silk cotton tree and sacred fig sapwoods come under the category of “very heavy attack (almost collapsed) to completely consumed”. However, mango, jujube tree, white siris, common guava, white leed-tree, shesham, long beak eucalyptus, white mulberry, black plum and banyan tree had “heavy attack (to penetration)” respectively. Sweet orange, litchi, neem, gum arabic tree, bamboo, mesquite, pongam oil tree, toothbrush tree suffered “superficial to medium attack (not deep inside)” respectively. The only wood species that suffered “slightly superficial attack” was white cedar (Table 2).

## Discussion

Efficiency of baiting in suppressing or eliminating the field colonies of termites can be improved by the selection of a suitable wood species, bait station size and bait placement. Wood species differ in density, nutrition and the amount and type of chemicals present in them (Scheffrahn, 1991). So to trap a large numbers of workers in the monitoring stations, palatability of the wood is of much importance. Highly palatable and soft woods attract more numbers of workers in the stations. The current study revealed the findings of preference of 21 native wood species to *M. mycophagus.*

Among the 21 wood species tested in the no-choice and choice tests in the current study, the sapwood of silk cotton tree was regarded as the most preferred wood species since *M. mycophagus* workers consumed the greatest mass from this wood. The silk cotton tree is a fast growing deciduous plant distributed throughout the tropics and sub tropics. Although, it has very stout and long trunk, it is not suitable for making timber structure because it is too soft to be used (Anonymous, 2014). However, softness and light weight properties of the silk cotton tree wood could be the reason it was highly preferred by *M. mycophagus*. There are studies which showed that termite prefers soft woods compared to hard woods which are usually difficult to chew and eat. Our results are in accordance with those of Peralta et al. (2004) who reported wood hardness as an important factor in wood consumption by subterranean termites with more consumption rates for softwood species compared to hardwood species. In other trails, Behr, Behr & Wilson (1972) and Bultman, Beal & Ampong (1979) found inverse relationship between wood hardness and wood feeding by *R. flavipes* and *Coptotermes formosanus* and reported that softer woods are more heavily damaged than heavier and harder woods.

Moreover, the wood of *Bombax* sp. is more vulnerable to degradation by the fungus (Mub, 2007) and termites usually prefer the woods that are degraded by the fungi (Little et al., 2013). On the other hand, white cedar was the least preferred wood species to *M. mycophagus* as indicated by the lowest mass loss in no-choice and choice trials. This could be due to the presence of different chemicals (e.g., limonoid analogues) in the sapwood (Huang & Chen, 1981) which had deterred workers of *M. mycophagus*. According to Scheffrahn (1991), wood resistance to termite attack depends upon the presence of chemicals in the lignocellulosic tissue. For the current study we did not determine the type and quantity of the chemicals in the sapwood of white cedar responsible for less mass loss but this could be important to find out such chemicals in the future.

### Conclusion and significance

Baiting studies are lacking in developing countries like Pakistan. This latest technique requires the use of some food materials that can attract a large numbers of workers into the bait stations. Current study revealed silk cotton tree and sacred fig as the most preferred wood species to *M. mycophagus*. These two woods can be efficiently utilized in trapping, monitoring and baiting program against *M. mycophagus*. This may increase the percentage of monitoring devices being attacked and aggregated by termites during the monitoring process. This may also decrease the cost of baiting since fewer visits to the baited site will be required of the pest control operator if the period between installation of monitoring stations and termite attack is reduced (Ngee et al., 2004).
